# Contributor Turnover Confounds Temporal Comparison of Recorded Habitats: A Voucher-Based Case Study of Three *Lycoris* Species in China from 2010 to 2020

**DOI:** 10.3390/plants15192933

**Published:** 2026-09-25

**Authors:** Lihui Mao, Yu Shen, Xuerui Cao, Qing Dong, Wei Hu, Danqing Tian, Kaimei Zhang, Qingcheng Zou

**Affiliations:** 1 Zhejiang Institute of Landscape Plants and Flowers, Zhejiang Academy of Agricultural Sciences, Hangzhou 311251, China; mlh@zaas.ac.cn (L.M.); caoxr@zaas.ac.cn (X.C.); dongq@zaas.ac.cn (Q.D.); huw@zaas.ac.cn (W.H.); tdq0123@163.com (D.T.); 2 College of Ecology and Environment, Nanjing Forestry University, Nanjing 210037, China; kaimeizhang@163.com

**Keywords:** *Lycoris*, Amaryllidaceae, herbarium records, recorded habitat, collection bias, institutional turnover, Chinese Virtual Herbarium, China

## Abstract

Temporal comparisons drawn from aggregated occurrence databases assume that the records compared were produced by a comparable community of collectors. We tested that assumption using 297 voucher-backed records of *Lycoris radiata*, *L. aurea*, and *L. chinensis* retrieved from the Chinese Virtual Herbarium, comparing 2010–2015 with 2016–2020. Recorded habitat composition changed; Forest/Woodland fell from 44.9% to 22.3% of coded records and Urban/Residential rose from 8.9% to 20.4% (χ^2^ = 20.308, df = 6, *p* = 0.002; permutation *p* = 0.002; Cramér’s V = 0.279). The contributing institutions and provinces, however, also fluctuated significantly (institution V = 0.550; province V = 0.380; both *p* < 0.001), and both were themselves strongly associated with recorded habitat. Stratification separated the two components: the Forest/Woodland decline persisted (Mantel–Haenszel χ^2^ = 8.574, *p* = 0.003 by province; χ^2^ = 6.250, *p* = 0.012 by institution), whereas the Grassland change did not (*p* = 0.871; *p* = 0.409). Record totals fell from 189 to 108, which we attribute to digitization lag rather than population change. Before interpreting temporal differences in aggregated occurrence data, researchers should examine changes in contributor composition and use stratified analyses to assess potential confounding.

## 1. Introduction

The genus *Lycoris* Herb. (Amaryllidaceae) comprises approximately 20 species of bulbous perennial herbs distributed mainly in warm-temperate and subtropical East Asia [1,2]. China contains the greatest recognized diversity of the genus, and areas south of the Yangtze River have long been regarded as a center of differentiation [1,2]. Several taxa are also sources of Amaryllidaceae alkaloids, including lycorine- and galantamine-related compounds, which has stimulated pharmacological interest in the genus [3,4]. These combined ornamental and pharmacological values make the status of wild and semi-natural *Lycoris* populations relevant to both basic plant science and applied conservation.

Biological interpretation within *Lycoris* is complicated by reticulate evolution. Hybridization, polyploidy, aneuploidy, variable fertility, and vegetative reproduction have contributed to difficult species boundaries and geographically structured cytotypes [3,4,5,6,7,8,9,10]. Chloroplast-genome comparisons and plastid sequence analyses have clarified several relationships but also revealed discordance expected under recurrent hybridization and maternal introgression [5,6,9,10]. Natural hybridization between *L. aurea* and *L. radiata* and the prolonged misidentification of hybrid taxa illustrate why occurrence records without vouchers can propagate taxonomic error [4,7]. For temporal distribution studies, taxonomic uncertainty can imitate ecological change when records are reassigned, differently identified, or compiled from maps produced under different identification standards.

The landscapes occupied by *Lycoris* are also changing. Land-use transformation is a major global driver of biodiversity loss, altering habitat amount, configuration, microclimate, and connectivity [11,12,13,14,15,16]. In China, recent decades have included cropland reorganization, urban expansion, forest recovery, ecological restoration, and substantial gross land-cover turnover [13,14,17,18]. A transition from open grassland or agricultural edges toward woodland, shrubland, infrastructure, or built environments may alter light availability, soil moisture, disturbance regimes, and the accessibility of populations to collectors. Conversely, persistent bulbs can survive short episodes of aboveground disturbance, and some geophytes occupy ecotones rather than a single closed vegetation type [19]. Broad vegetation categories therefore provide useful context but cannot by themselves establish habitat suitability.

Climate change provides an additional but interacting source of distributional change [20,21]. Poleward and upward range shifts are widely reported across taxa [22,23,24], and climate-driven changes in water availability and agriculture are especially relevant in China [25]. Modeling of the *L. aurea* complex suggests that temperature and precipitation may shape both historical distributions and future suitability, potentially with lineage- or cytotype-specific responses [26]. Nevertheless, an apparent movement in a set of occurrence records cannot automatically be attributed to climate. Landscape conversion, uneven search effort, changes in site accessibility, and the availability of records from range margins can alter the apparent center or extent of the recorded distribution without corresponding shifts in plant populations.

Occurrence data are indispensable for documenting plant distributions, but their inferential limits are substantial. Herbarium, literature, citizen-science, and map-derived data often have spatial, temporal, and taxonomic biases [27,28,29,30,31,32]. Collection efforts are concentrated near roads, settlements, institutions, and accessible protected areas, and the intensity of collecting can vary sharply among years [27,28]. Coordinate errors or values recovered from image axes may be mistaken for geographic coordinates, while duplicated or clustered localities can dominate descriptive summaries [29,30,32]. These issues matter most in small datasets, because where only a few hundred records are available, a handful of them can alter species proportions, habitat counts, range limits, or model outputs [33].

Herbarium specimens are not a legacy archive but an active research infrastructure. Voucher deposition is now expected, and in much of the taxonomic and ecological literature, it is required for published distributional and taxonomic claims, and specimen citations are increasingly part of how such claims are verified. In parallel, the digitization of Chinese herbarium holdings has made the accumulated record searchable at a scale that did not exist a decade ago [34,35]. The consequence for analysis is not only that more records are available, but that they were produced for very different reasons. A dedicated survey of a single species, a regional flora inventory, an opportunistic record made in passing, and material collected for the ornamental or medicinal bulb trade all enter the same aggregated database, and the balance among them shifts as funding programs, institutional priorities, and collector interests change. As voucher-based temporal studies become more common, they will increasingly need to account for differences in collection purpose and changes in the institutions and collectors contributing records. The analysis described below provides a straightforward way to assess whether changes in contributing institutions and collection regions may explain the observed temporal differences.

This study is an exploratory, hypothesis-generating case study of one such dataset. Its object is a single question: when the recorded habitat composition of an occurrence dataset changes between two periods, how much of that change is attributable to a change in who contributed the records? We assembled 297 herbarium records of *L. radiata*, *L. aurea*, and *L. chinensis* collected between 2010 and 2020, retrieved from the Chinese Virtual Herbarium [34], and compared the periods 2010–2015 and 2016–2020. The approach uses only standard contingency-table statistics, including Pearson’s chi-square test, Cramér’s V, and Mantel–Haenszel stratified tests [33,36]. Its validity rests on the assumptions of those tests; no new method is claimed. The phenomenon it addresses is not new; the confounding effect of observer or contributor identity on temporal comparison is documented both for structured monitoring [37] and for aggregated occurrence data [27,28,29,30,31,32,38]. To our knowledge, few studies have explicitly examined how accounting for contributor turnover affects temporal comparisons of recorded habitats in small, voucher-backed datasets. We address this question using the present dataset; this paper supplies an estimate. This type of check has three uses. The analysis can be performed before interpreting temporal differences, using institution and locality metadata available in the occurrence records. For monitoring, it shows where new fieldwork would be most informative: the georeferenced localities that can be revisited, and the provinces that dropped out of the later record set. As digitized herbarium datasets expand, this analysis can help reduce the risk of interpreting changes in collection activity as changes in plant distributions. We first compared record totals, species composition, contributing institutions, collection provinces, and recorded habitat composition between periods. We then tested whether differences in recorded habitat remained significant after stratification by institution or province.

Two adjacent five-year windows were chosen deliberately. Comparisons on this timescale are in current use, including the analysis whose habitat classification we adopt [35], and they are the interval over which a stable community of contributors is most often assumed. Over a longer interval, digitization of herbarium holdings, changes in collecting practice and taxonomic revision all accumulate alongside the turnover we wish to measure, and the two cannot then be separated. Restricting the comparison to two adjacent five-year windows keeps these other sources of change small. Recorded habitat composition may change because plant distributions change or because collectors and institutions sample different locations. Examining contributor composition is therefore necessary when interpreting temporal differences.

## 2. Results

### 2.1. Composition of the Dataset

The dataset contained 189 records from 2010–2015 and 108 from 2016–2020 (Table 1). These totals describe the records available at retrieval and should not be interpreted as estimates of plant abundance or population change.

Despite the differing percentage changes, species composition was statistically indistinguishable between periods (χ^2^ = 3.258, df = 2, *p* = 0.196; Cramér’s V = 0.105, no expected count below five), and the same held after collapsing gatherings (χ^2^ = 3.046, df = 2, *p* = 0.218). The proportional contribution of *L. radiata* was 59.8% and 53.7% in the two periods, that of L. aurea was 33.3% and 42.6%, and of L. chinensis was 6.9% and 3.7%. The recorded decline was therefore broadly proportional across taxa rather than concentrated in one species, and the apparently severe fall in *L. chinensis* rests on 13 and 4 records and cannot be distinguished from sampling noise (Figure 1).

### 2.2. Turnover in Contributing Institutions and Provinces

The set of institutions contributing records changed markedly between periods. The period × institution association across all 30 herbaria was strong and highly significant (χ^2^ = 89.900, df = 29, *p* < 0.001; permutation *p* < 0.0001; Cramér’s V = 0.550; Table 2), and it remained so when the five largest contributors were compared against all others combined (χ^2^ = 25.734, df = 5, *p* < 0.001; Cramér’s V = 0.294), a grouping in which no expected count fell below five. Contribution was also highly uneven overall; the single largest herbarium supplied 55 of 297 records (18.5%), and the five largest supplied 151 (50.8%). Counts by herbarium are given in Table S2.

The geographic distribution of records shifted in parallel (χ^2^ = 42.818, df = 16, *p* < 0.001; permutation *p* = 0.0001; Cramér’s V = 0.380). Guizhou contributed 33 records in each period and Guangxi 36 and 28, whereas Jiangxi fell from 27 to 11, Hunan from 24 to 6, Hubei from 20 to 4, and Sichuan from 12 to 2; Shanghai, Jiangsu, and Gansu contributed 6, 4, and 4 records in the earlier period and none in the later one. The later dataset contained fewer records and a greater proportion of records from southwestern China (Figure 2; Table S3). 

Because recorded habitat composition differs among herbaria and provinces, changes in their relative contributions can alter the overall habitat proportions without any change in plant distributions. The stratified analysis assesses whether temporal differences remain after accounting for these changes.

### 2.3. Recorded Habitat Composition

Of the 297 records, 261 carried a habitat description and were coded (158 from 2010–2015 and 103 from 2016–2020). Forest/Woodland was the most frequent recorded habitat in both periods but fell sharply, from 71 of 158 coded records (44.9%) to 23 of 103 (22.3%), a decrease of 22.6 percentage points (Table 3; Figure 3a). Urban/Residential rose from 14 records (8.9%) to 21 (20.4%), an increase of 11.5 points. Grassland rose from 23 (14.6%) to 21 (20.4%) and Shrubwood/Sparsewood from 15 (9.5%) to 13 (12.6%). Occupancy of the Other category was essentially unchanged (20.3% versus 21.4%), while Dry Land and Paddy together accounted for fewer than 3% of records in either period. Counts by species are given in Table S4.

The period × habitat association across all seven classes was significant (χ^2^ = 20.308, df = 6, *p* = 0.002; permutation *p* = 0.002; Cramér’s V = 0.279), although 4 of 14 expected counts fell below five. Collapsing to three classes removed the sparsity and preserved the result (χ^2^ = 13.829, df = 2, *p* < 0.001; Cramér’s V = 0.230, no expected count below five). In separate binary comparisons, the proportion of Forest/Woodland records differed significantly between periods (χ^2^ = 13.828, *p* < 0.001), whereas the proportion of Grassland records did not (χ^2^ = 1.513, *p* = 0.219). All tests are summarized in Table S6.

### 2.4. Confounding by Institution and Province

Contributing institution and province were themselves strongly associated with recorded habitat. The institution × habitat table gave χ^2^ = 349.318, df = 138, *p* < 0.001 (permutation *p* = 0.0001; Cramér’s V = 0.472) and the province × habitat table χ^2^ = 341.224, df = 90, *p* < 0.001 (permutation *p* < 0.0001; Cramér’s V = 0.467). Although both tables were sparse, the observed associations suggest that recorded habitat composition differed among contributing herbaria and provinces.

Under stratification the two habitat contrasts behaved differently. The Forest/Woodland decline persisted when the comparison was stratified by province (Mantel–Haenszel χ^2^ = 8.574, df = 1, *p* = 0.003; Table 2) and by contributing institution (χ^2^ = 6.250, df = 1, *p* = 0.012), in both cases attenuated relative to the unstratified statistic (χ^2^ = 13.828) but still significant. The Grassland contrast, which was already non-significant unstratified, fell to χ^2^ = 0.027, *p* = 0.871 stratified by province and χ^2^ = 0.683, *p* = 0.409 stratified by institution. The lower proportion of Forest/Woodland records in the later period remained significant after stratification by institution or province. The Grassland contrast was not significant either before or after stratification (Figure 3b).

### 2.5. Sensitivity to Duplicate Gatherings

Repeating the analyses on the duplicate-collapsed dataset (*n* = 209) left the direction of every effect unchanged. Record totals fell from 131 to 78, species composition remained statistically unchanged (χ^2^ = 3.046, df = 2, *p* = 0.218), and the direction and approximate magnitude of the habitat and contributor effects were preserved. On the collapsed dataset, however, the seven-class period × habitat association, computed on the 178 coded records that survive collapsing, is no longer significant (χ^2^ = 9.371, df = 6, *p* = 0.154; Cramér’s V = 0.229, with 4 of 14 expected counts below five), and neither is the Forest/Woodland contrast taken alone (χ^2^ = 2.173, *p* = 0.140), although both effect sizes remain close to those computed on the full dataset (Table S6). Because collapsing removed 88 records, the collapsed tests had lower power, and they are reported as a robustness check rather than as the primary analysis.

## 3. Discussion

### 3.1. The Record Decline and Its Likely Causes

Three patterns emerge. First, the total number of available records decreased by 42.9%, with fewer records for all three species in the later period. Record totals also declined after duplicate gatherings were collapsed. Second, the composition of contributing institutions and provinces changed strongly over the same interval. Third, recorded habitat composition shifted away from Forest/Woodland and toward Urban/Residential, and the Forest/Woodland component of that shift survived stratification on both contributor variables.

The decline in recording itself is the pattern least likely to be biological. Herbarium datasets accumulate with a lag; specimens collected recently are less likely to have been mounted, accessioned, databased, and published to an aggregator by the time of retrieval [27,28]. The annual record counts in this dataset peaked in 2014 and 2015 and fell thereafter, which is exactly the shape that digitization lag produces. A 42.9% fall in retrievable records across a five-year boundary therefore measures the availability of evidence rather than the size of the populations. The same reasoning applies to the disappearance of Shanghai, Jiangsu, and Gansu from the later period. These provinces contributed four to six records each, and their absence is more parsimoniously read as the cessation of one or two collectors’ activity than as local extirpation.

That species composition did not change is itself informative. If the decline were driven by a taxon-specific process, for example, targeted bulb collection of *L. radiata* or habitat loss concentrated in the range of one species, the proportional contributions would be expected to diverge. They did not (*p* = 0.196), which is consistent with a shared, non-biological cause acting on all three species at once. The apparent 69.2% fall in *L. chinensis* rests on a change from 13 to 4 records and should not be interpreted; with counts this small, the confidence interval on the proportional change spans most of the plausible range.

None of this excludes biological contribution. Habitat conversion, infrastructure development, agricultural intensification, and bulb collection could all remove accessible populations, and many widely naturalized triploid forms of *L. radiata* reproduce predominantly vegetatively, which may slow recolonization after local removal [5,8,9]. Persistent bulbs buffer short disturbance episodes but do not guarantee recovery after excavation, soil sealing, or repeated vegetation management [19]. This dataset cannot distinguish biological changes from changes in collecting and digitization. Standardized resurveys of documented localities, combined with occupancy analyses, could help distinguish these explanations.

### 3.2. Interpreting the Habitat Shift

The main habitat-related finding was a lower proportion of Forest/Woodland records in the later period, which remained significant after stratification by province or institution. Forest/Woodland fell from 44.9% to 22.3% of coded records, while Urban/Residential rose from 8.9% to 20.4%, and the Forest/Woodland decline survived stratification on province (*p* = 0.003) and on contributing institution (*p* = 0.012). The Forest/Woodland contrast remained significant after stratification by province or institution, although the test statistics were smaller than in the unstratified analysis.

Two explanations are consistent with the temporal difference remaining after stratification. Collectors may have increasingly sampled accessible sites, such as roadsides, village margins, and managed green spaces. This shift could increase the proportion of Urban/Residential records and decrease the proportion of Forest/Woodland records without any change in plant distributions. Alternatively, forest-associated occurrences may genuinely have become harder to find. These alternatives are not separable here, and the first is the more parsimonious given the strong accessibility bias documented for opportunistic collections generally [27,28,29,32,39]. The change therefore reflects more than which herbaria and which provinces happened to contribute.

The results reported here differ in strength and should be read accordingly. The institution × habitat and province × habitat tables are sparse (155 of 168 and 96 of 112 expected counts below five), so they are descriptive only; they estimate the magnitude of an association and support no inferential claim. They are reported because that magnitude is what motivates the stratified analysis that follows. The period × habitat and period × contributor tables are also sparse in places, but permutation tests make no asymptotic assumption and we treat their *p*-values as informal. The conclusion is based primarily on the Mantel–Haenszel tests using stratified 2 × 2 tables. The Mantel–Haenszel tests assume a common association across strata, which was not formally testable here given stratum sizes. The habitat coding was carried out by a single coder against a rubric fixed in advance, and although the direction of the Forest/Woodland change is too large to be plausibly reversed by recoding, the exact percentages will move.

Ecologically, a woodland–ecotone association is plausible for these taxa. Edges and openings in woodland moderate temperature and moisture, while open sites provide seasonal light after canopy senescence, and several *Lycoris* species occupy such transitional positions rather than a single closed vegetation type [19]. The recorded habitat classes are broad: Forest/Woodland here spans closed forest, plantations, edges, secondary woodland, and understory, and Urban/Residential spans roadsides, village margins, and managed plantings. Site-level measurement of canopy cover, soil moisture, disturbance, and management would be required to convert these categories into statements about habitat requirement.

Coding decisions affect effect sizes and not only percentages, and the magnitude is worth stating. Reviewing the coding against the rubric in Table S5 identified 16 records whose original assignment did not follow it. Applying the rubric strictly to those records attenuates every statistic reported above, by 8% for the seven-class association and by a fifth to a third for the Forest/Woodland contrasts. The seven-class period × habitat association falls from χ^2^ = 20.308 (*p* = 0.002, V = 0.279) to χ^2^ = 18.689 (*p* = 0.005, V = 0.268); the Forest/Woodland contrast from χ^2^ = 13.828 (V = 0.230) to χ^2^ = 9.420 (V = 0.190); the province-stratified Mantel–Haenszel statistic from 8.574 (*p* = 0.003) to 6.300 (*p* = 0.012); and the institution-stratified statistic from 6.250 (*p* = 0.012) to 4.995 (*p* = 0.025). Under the corrected coding, both stratified Forest/Woodland contrasts remained significant, whereas the Grassland contrast remained non-significant. However, the institution-stratified *p*-value increased from 0.012 to 0.025, indicating that the statistical evidence was sensitive to coding decisions.

### 3.3. Contributor Turnover as a General Problem

This study illustrates the importance of examining contributor turnover when interpreting temporal differences in aggregated occurrence records. Between two adjacent five-year windows, the composition of contributing herbaria changed with an effect size of V = 0.550 and the composition of the provinces from which records originated with V = 0.380, while both variables were themselves strongly associated with the outcome of interest. Any period comparison computed from these records without examining those two variables would have reported the habitat shift at more than one and a half times its stratified magnitude.

This is not specific to *Lycoris* or to herbarium data. Aggregated occurrence datasets are assembled from contributors whose activity starts and stops, and whose collecting is spatially and institutionally structured. Where a temporal contrast is the object of study, the identity of the contributors is a candidate confounder in the same way that observer identity is in structured monitoring. The check is inexpensive and can be stated as a procedure. First, cross-tabulate period against contributing institution and period against region, and report both tables. Second, if either association is substantial, test whether the contributor variable is itself associated with the outcome. Third, where it is, repeat the temporal contrast under stratification and report the stratified statistic alongside the unstratified one, so that a reader can see how much of the contrast the contributors account for. None of this requires data beyond fields that aggregated occurrence databases already carry. In the present dataset, it reduced the Forest/Woodland contrast by roughly a third and the Grassland contrast was not significant before or after stratification. We therefore suggest that any temporal comparison of aggregated occurrence data should report two cross-tabulations as standard practice: period × contributing institution, and period × region.

### 3.4. Taxonomic, Provenance, and Conservation Implications

*Lycoris* taxonomy adds a further layer of uncertainty. Hybridization and reticulate evolution can cause morphologically similar plants to be determined differently by different specialists [3,4,5,6,9], and the description of *L. × jinzheniae* after three decades of misidentification shows that apparently stable names on herbarium sheets may be revised [7]. Determinations here were accepted as recorded and were not revisited. Because the three study species differ in flower color and are not usually confused with one another, this is unlikely to have driven the period contrasts reported above, but it does mean that species-level totals should be read as determinations on record rather than as verified identifications. Chloroplast genomes and microsatellite markers provide tools for verification where it matters [6,8,10].

Each record includes an institution code, catalog number, collector name, and collection date, together with an explicit coordinate-status flag. These metadata allowed us to assess potential confounding by contributing institution and collection province. The remaining gaps are the absence of a coordinate-uncertainty ratio, the absence of any statement of how each coordinate was obtained, and the 83 records with no usable position. Recording coordinate uncertainty and georeferencing methods would provide a clearer basis for linking occurrence records to environmental layers [30].

For monitoring, the dataset supports modest and specific priorities. The 214 georeferenced records provide a starting point for planning resurveys. Provinces with marked declines in record numbers, including Jiangxi, Hunan, Hubei, and Sichuan, warrant particular attention. Revisiting documented localities in these provinces could help determine whether the lower record counts reflect changes in collecting activity, incomplete digitization, or changes in plant occurrence. Surveys should distinguish failure to relocate a population from failure to detect it, record management and canopy context directly rather than inferring them from a label, and retain vouchers.

The same surveys could also expand the occurrence inventory for *L. chinensis*. With only 17 records across the study period, the available dataset provides insufficient information for reliable temporal inference for this species. Plant diversity and endemism hotspots in China overlap areas under substantial land-use pressure [40], and narrow-range taxa can be vulnerable even when short time-series counts appear stable or, as here, are simply too sparse to read. *L. aurea* should be monitored with attention to hybridization and cytotype, since climatic response may vary within the species complex [4,26].

### 3.5. Limitations and Future Research

The study has several central limitations. First, the records are opportunistic; they carry no measure of search effort and no repeated visits, so counts index available evidence rather than occurrence. Second, herbarium digitization lag almost certainly contributes to the apparent decline, and its magnitude cannot be estimated from the data themselves. Third, habitat classes were coded from collectors’ free-text notes, which describe the immediate surroundings of a plant rather than a mapped land-cover class. The coding was performed by a single author against a rubric fixed in advance, and no independent second coding was carried out, so no inter-coder agreement statistic can be reported. Readers who wish to assess the coding directly can do so from Excel Table S1, which releases every original description alongside its assigned class. Several contingency tables are sparse, particularly those involving institutions and province, which limits asymptotic inference. Meanwhile, 83 records lacked usable coordinates, and none carried an uncertainty estimate, which precluded any connection to environmental data. Sixth, the dataset cannot support species-level inference: with 17 records of *L. chinensis* across the decade and sparse habitat tables for all three species, the species-level tables in Table S4 are released for audit rather than for interpretation. Finally, the purpose for which each specimen was collected was not recorded in the portal. Collecting motivation is heterogeneous; reasons include a dedicated survey of one species, a regional flora inventory, an opportunistic record made in passing, or material taken for the ornamental or medicinal bulb trade. Purposes may differ as funding programs and institutional priorities change, so their contribution to the change in recorded composition cannot be separated from digitization lag in these data.

Several additional features of the dataset limit interpretation. Almost half of the records (48.5%) belonged to duplicate gatherings, and 12% lacked a habitat description. Contribution was highly concentrated, with one herbarium supplying 18.5% of all records. Stratified analysis reduces confounding by contributor but cannot remove bias shared across contributors, such as the general tendency to collect near roads and settlements, nor does it correct for what never entered the portal. The dataset was drawn from holdings that have been digitized and published, and the herbaria that have done so most completely are not a random subset of those holding *Lycoris* material.

A stronger next phase would combine three elements. Standardized resurvey of the 214 georeferenced localities with fixed effort would allow detection and occupancy to be separated. Coordinate-uncertainty estimates, obtainable by re-georeferencing the locality text under an explicit protocol [28], would permit the recorded habitats to be checked against annual 30 m land-cover products [18] and against climate surfaces [41], converting the collector’s field impression into a testable proposition. Where distribution models are used, they should be tuned rather than accepted at default complexity, evaluated on spatially separated data, and fitted with effort covariates or target-group backgrounds to address the accessibility bias documented here [28,29,32,42,43,44,45,46,47,48,49].

A formal validation of the check would require either a reference dataset in which the true underlying habitat is known independently of the collectors’ labels, or a simulation in which contributor turnover is imposed at a known strength, so that the sensitivity of the check can be calibrated. Neither is available here, and we therefore present the check as a screening step rather than as a calibrated method.

Finally, the record-level table and the habitat-coding rubric are released together so that every number in this article can be regenerated and every coding decision audited. The confounder analysis that changed the interpretation of the habitat result required nothing more than two additional cross-tabulations of fields that were already present in the retrieved records. These results highlight the value of examining contributor composition before interpreting temporal differences in aggregated occurrence records.

## 4. Materials and Methods

### 4.1. Study Design and Scope

We analyzed a single dataset of 297 voucher-backed occurrence records for three *Lycoris* species, assigned to 2010–2015 or 2016–2020 from the collection date on the specimen label. The study unit was an individual herbarium record, carrying species determination, institution code, catalog number, collection date, collector, province, county or district, locality text, elevation where available, coordinates where available, and the collector’s free-text description of the habitat. Every quantity reported below is a count or a proportion of such records.

The workflow was retrospective and observational. Specimens accumulate through unstandardized collecting efforts, so the dataset carries no measure of search effort, detection probability, or repeated sampling at fixed sites. Accordingly, terms such as increase and decrease refer throughout to counts of records, not to numbers of plants or populations, and this distinction is maintained in figure captions, table notes, and the interpretation of every test reported below [27,31].

The findings reported below are therefore properties of the retrieved record set and are stated as such. Recorded habitat composition differed between the two periods, the set of contributing institutions and provinces also differed, and the Forest/Woodland component of the habitat difference survived stratification of those contributors. Each of these statements can be checked directly against the released record-level table (Excel Table S1).

### 4.2. Study Species

The analysis included *L. radiata* (L’Hér.) Herb., *L. aurea* (L’Hér.) Herb., and *L. chinensis* Traub (Figure 4). *L. radiata* is widely recorded in China and includes cytologically and reproductively variable populations; some common triploid forms reproduce predominantly vegetatively [5,8,9]. *L. aurea* is concentrated mainly in southern China and participates in natural hybridization with related taxa [4]. *L. chinensis* has a comparatively restricted documented distribution in central-eastern China [1,2]. Species determinations were accepted as recorded by the contributing herbaria and were not independently revised. Reticulate evolution introduces uncertainty in both species-level record totals and comparisons between periods. The three study species differ conspicuously in perianth color and form and are not routinely confused with one another, so determination error is unlikely to move records between them in a pattern capable of generating the period contrasts below. The residual risk is of a different kind; a hybrid or an unrecognized segregate may be filed under one of the three names, as happened for three decades with *L. × jinzheniae* [7]. Such errors may affect species-level record totals. Because identifications were not independently verified, we cannot exclude their influence on comparisons between periods.

### 4.3. Record Retrieval and Period Assignment

Records were retrieved from the Chinese Virtual Herbarium (CVH), the national specimen data portal maintained by the Institute of Botany, Chinese Academy of Sciences [34], on 20 November 2025. The search was restricted to *Lycoris radiata*, *L. aurea*, and *L. chinensis*, to physical specimen records rather than image-only or literature entries, and to collection dates between 5 September 2010 and 20 September 2020; records annotated as cultivated or as originating from botanical-garden or nursery material were excluded. Each retained record was assigned to 2010–2015 or 2016–2020 from its collection date, and no record was removed after retrieval. Every record retains its original portal identifier alongside a stable identifier assigned in the released table.

Records sharing collector, collection date, and locality were flagged as belonging to a single gathering [30]. Such records are predominantly herbarium duplicates of one collecting event distributed among several institutions, not independent observations, and treating them as independent would overstate both the sample size and any apparent concentration of effort. In total, 144 records (48.5%) fell into 56 gatherings. All reported counts retain the flagged records, and every test was repeated on a collapsed dataset in which each gathering contributes one record (*n* = 209) as a sensitivity analysis. The record was retained as the primary unit for two reasons. The quantities compared here—which institution holds a specimen, which province it came from, what habitat the collector recorded—are properties of records, and duplicates of one gathering distributed among several herbaria are part of the contributor structure under study rather than noise to be removed from it. Because duplicate specimens are not independent observations, we repeated all tests using one record per gathering to assess the sensitivity of the results to duplication. Collapsing removes 88 of the 297 records and reduces power correspondingly.

The retained inventory comprised 189 records from 2010–2015 and 108 from 2016–2020, contributed by 30 herbaria and 145 collectors and distributed across 17 provincial-level units. Both sets of contributors turned over substantially between periods. The number of contributing herbaria fell from 27 to 19, with 16 active in both periods, 11 in the earlier period only, and 3 in the later period only. Among collectors, the turnover is close to complete: 92 individuals or teams contributed in the earlier period and 59 in the later one, and only 6 of the 145 collectors in the dataset appeared in both. Collection dates spanned 5 September 2010 to 20 September 2020, and elevation was recorded for 249 of the 297 records. Contributions by herbarium and by collection year are listed in Tables S2 and S7. Full details are given in Methods S1.

### 4.4. Coordinate Quality and Spatial Visualization

Coordinates were retained only where the label values could be parsed to decimal degrees. Of the 297 records, 208 carried coordinates recorded directly in decimal degrees and 6 were converted from degrees–minutes–seconds, giving 214 usable positions; 80 were missing, 2 were partial, and 1 was a 0,0 placeholder. The released table specifies the coordinate status of each record, distinguishing usable coordinates from missing, incomplete, or placeholder values [30]. All 214 positions fell inside the terrestrial bounding box of China, and each was checked against the administrative unit named on the label; no record was placed in a province inconsistent with its locality text. Coordinate status by species is given in Table S8. The records carried no coordinate-uncertainty radius and no statement of how each position was determined; so, positional error remains unquantified.

Coordinates were used only to display the recorded localities and to describe their provincial distribution. Figure 2 therefore shows the 214 georeferenced records only, 126 from 2010–2015 and 88 from 2016–2020, and not the full set of 297; the 83 records without a usable position are included in every count and test reported below but cannot be mapped. The base map used for Figure 2 was obtained from the Standard Map Service of the Ministry of Natural Resources of China (http://bzdt.ch.mnr.gov.cn/, (accessed on 20 November 2025), approval number GS(2019)1682, scale 1:22,000,000; national, provincial, and maritime boundaries, the South China Sea islands inset, and the approval statement were reproduced as supplied and have not been edited, and only the occurrence symbols and panel labels were added.

### 4.5. Habitat Coding

Collector habitat descriptions are free text. The 261 records carrying a description comprised 133 distinct strings, ranging from single terms such as shrubland to compound descriptions such as broadleaved forest; forest understory; calcareous soil. These 133 descriptions were coded into seven classes, Grassland, Forest/Woodland, Shrubwood/Sparsewood, Urban/Residential, Dry Land, Paddy, and Other, so that every test reported below was computed on a seven-class table, not on the descriptions themselves. These are the classes of the national land-use classification of China and were adopted here, rather than defined for this study, so the results remain comparable with an earlier five-year analysis of distribution change in China that used the same scheme [35]. The definition applied to each class is given in Table 4; the full rubric, which states six ordered rules of application together with verbatim representative source phrases and their record counts, was agreed before coding began and is reproduced as Table S5. The coding was carried out by one author. No independent second coding was performed, and no inter-coder agreement statistic is therefore available. Excel Table S1 provides all 261 original habitat descriptions and their assigned classes, allowing readers to independently review the coding of the 133 distinct descriptions and repeat the habitat analyses. The 36 records without a habitat description were excluded from habitat analyses, leaving 261 coded records; percentages for habitat classes therefore use 158 and 103 as period denominators. Field definitions for the released table are given in Table S9.

A collector’s habitat note is a field impression recorded at the moment of collection. It describes the immediate surroundings of the plant and is therefore informative about the vegetation context in which each specimen was found. Because it is not a land-cover classification, the seven classes were adopted as a fixed vocabulary for coding the collectors’ text (Table 4). The coding rules, the original descriptions, and the classes assigned are all released (Tables S1 and S5), so that the coding can be audited and, if necessary, revisited. Habitat-class names are capitalized when they are used as labels for the seven defined classes of Table 4 and are lower case when the same words are used generically (for example, “broadleaved forest”, “forest edge”, “urban expansion”); they are the defined class labels of the national land-use classification and are capitalized as defined terms rather than as common nouns.

### 4.6. Statistical Analysis

For each species and period, we calculated record count and proportional contribution to the period total. Habitat composition was summarized as *n* and percentage within period. Pearson chi-square tests of independence were used for period × species, period × province, period × contributing institution, and period × coded habitat [33]. Cramér’s V was used as the effect-size index and was calculated as V = √(χ^2^/[*n*(k − 1)]), where χ^2^ is the Pearson chi-square statistic for the table, *n* is the number of records contributing to that table, and k is the smaller of the table’s two dimensions, that is, the smaller of the number of rows and the number of columns. The number of records contributing to an analysis differs between tests and is stated with each result: 297 for the tests involving species, province and contributing institution; 261 for the habitat analyses, since 36 records carry no usable habitat description; 209 for the duplicate-collapsed sensitivity analysis; and 158 and 103 for the two period columns of the habitat table. Significance was evaluated at α = 0.05, but every *p*-value is reported together with the cell counts and the number of expected counts below five, because several tables are sparse. A three-class habitat grouping (Forest/Woodland, Grassland, Other) was analyzed alongside the full seven-class table as a sensitivity analysis.

Because contributing institutions and provinces changed between periods, a change in recorded habitat could arise from a genuine change in where plants were found or simply from a change in who was collecting and where. To separate these, we first tested whether institution and province were themselves associated with recorded habitat and then repeated the period × habitat comparison under stratification. Mantel–Haenszel tests [36] were computed for Forest/Woodland versus all other classes, and for Grassland versus all other classes, stratifying in turn by province and by contributing institution. A temporal association that remains significant after stratification suggests that the contributor variable does not fully explain the observed difference. Attenuation after stratification may indicate confounding, although changes in statistical power must also be considered.

All statistical analyses and all figures were produced in R (4.6.1; R Core Team) [50]. Chi-square tests, Cramér’s V, and the Mantel–Haenszel statistics were computed from the record-level table released with this article, and no result reported below was transcribed from an intermediate summary.

Because several tables are sparse, every chi-square test computed on a record-level table containing an expected count below five was repeated as a Monte Carlo permutation test with 20,000 replicates, and the permutation *p*-value is reported alongside the asymptotic one in Table 2 and Table S6. The duplicate-collapsed tests are reported as a robustness check with asymptotic values only. Strata that fall entirely within one period or one habitat category contribute zero variance to a Mantel–Haenszel statistic and do not enter the test; for the Forest/Woodland contrast, 9 of 17 provinces and 13 of 30 contributing institutions are informative in this sense, and these counts are reported with the statistics.

### 4.7. Reproducibility and Reporting

All numerical results reported in this manuscript were regenerated from a single record-level file released as Excel Table S1. That table preserves the record identifier, the original portal identifier, species, period, institution code, catalog number, collection date, collector, province, county or district, locality text, elevation, decimal coordinates where available, an explicit coordinate-status flag, the collector’s original habitat text, the coded habitat class, the gathering identifier, and a concise data-quality note. No record was removed. Reporting follows the principle that descriptive changes in opportunistic records should not be presented as population trends [27,28,29,30,31].

## 5. Conclusions

Between 2010–2015 and 2016–2020, the recorded habitat composition of voucher records for three *Lycoris* species in China changed, with Forest/Woodland falling from 44.9% to 22.3% of coded records and Urban/Residential rising from 8.9% to 20.4%. Over the same interval, the contributing herbaria and provinces differed significantly, and both of these were themselves strongly associated with recorded habitat. The Forest/Woodland contrast remained significant after stratification by province and by institution, whereas the Grassland contrast was not significant before or after stratification. The number of retrievable records fell from 189 to 108 over the same interval, proportionally across all three species and robustly to the removal of duplicate gatherings; this is most parsimoniously attributed to digitization lag and shifting collector activity, and no population inference is drawn from it.

The broader conclusion is procedural; where a temporal contrast is computed from aggregated occurrence records, the identity of the contributing institutions and regions is a candidate confounder that should be tabulated and, where substantial, stratified upon, before the contrast is interpreted. Monitoring priorities include standardized resurveys of documented localities in provinces with marked declines in record numbers and expanded occurrence surveys for *L. chinensis*. These surveys should document sampling effort and coordinate uncertainty.

These two priorities are complementary rather than alternative. The 214 georeferenced records can guide the selection of resurvey sites, with particular attention to Jiangxi, Hunan, Hubei, and Sichuan, where record numbers declined markedly. Standardized field surveys could help assess whether the lower proportion of Forest/Woodland records reflects changes in plant occurrence or differences in collection coverage. *L. chinensis*, with 17 records across the whole decade, requires expanded inventory before any such assessment is possible for that species at all. Standardized resurveys should use repeated visits to account for imperfect detection, directly record management conditions and canopy cover, and retain voucher specimens. These surveys could assess the observed habitat differences while expanding baseline information for the poorly represented *L. chinensis*.

## Figures and Tables

**Figure 1:**
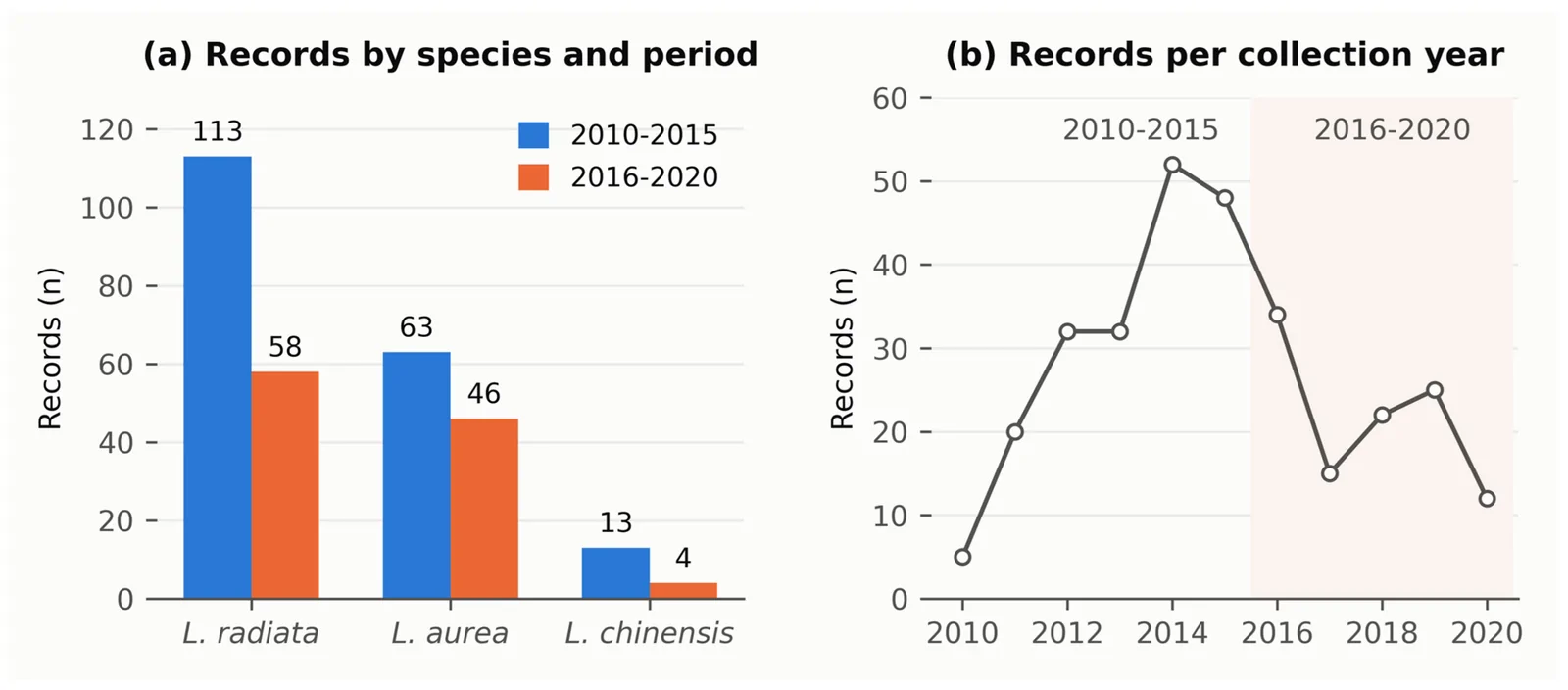
Voucher occurrence records of the three *Lycoris* species. (**a**) Records by species and study period. (**b**) Records by collection year, with 2016–2020 shaded. Counts index available records, not abundance, occupancy, or population size; the peak in 2014–2015 and the subsequent fall are consistent with herbarium digitization lag, because recently collected specimens are least likely to have been accessioned and published to an aggregator at the time of retrieval.

**Figure 2:**
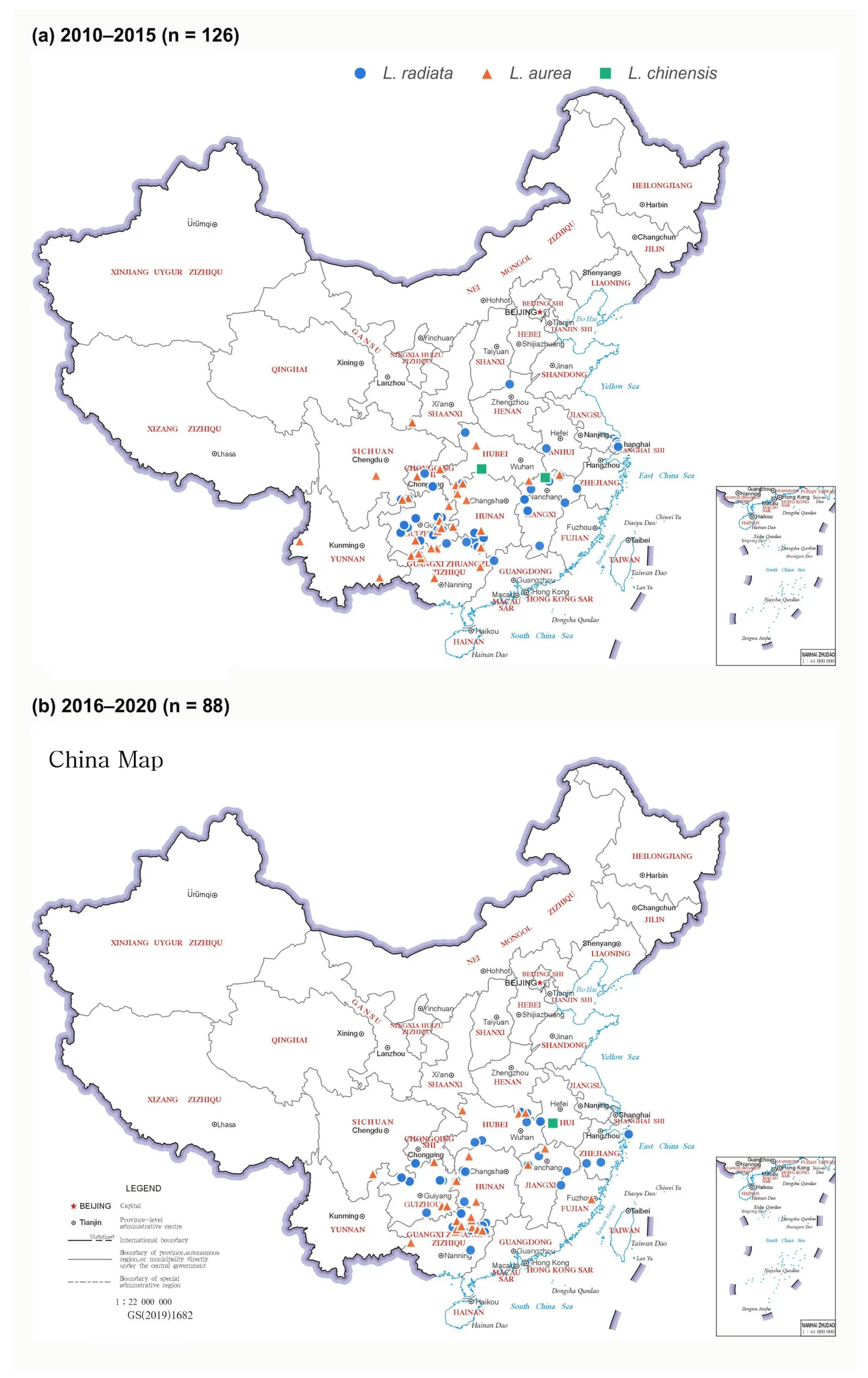
Recorded localities of *L. radiata*, *L. aurea*, and *L. chinensis* in (**a**) 2010–2015 and (**b**) 2016–2020. Symbols show the 214 of 297 voucher records that carry a usable coordinate (126 and 88 in the two periods); the 83 records without a usable position are included in all counts and tests but cannot be mapped. Species are distinguished by both color and symbol shape so that the panels remain legible in grayscale.

**Figure 3:**
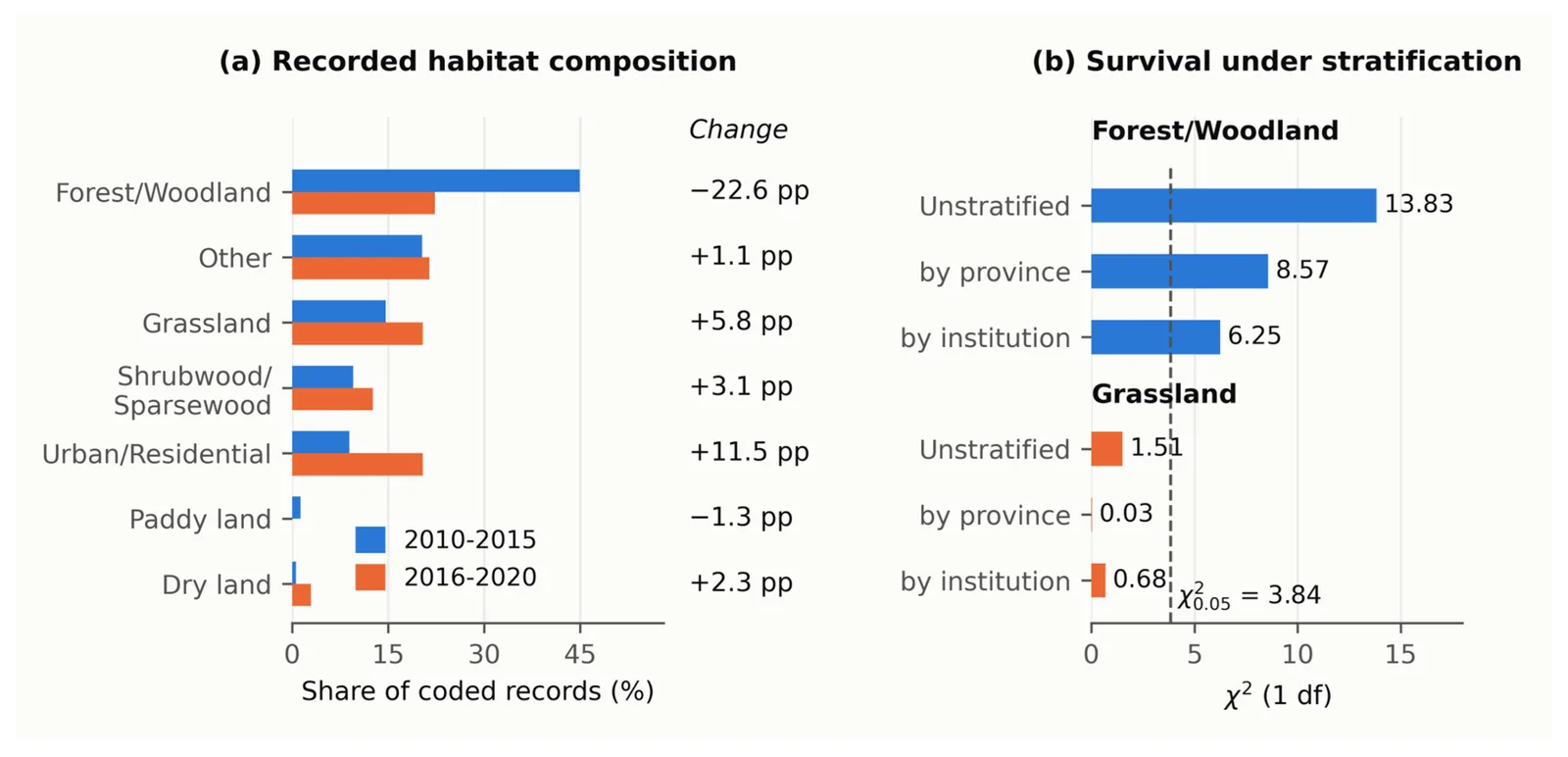
Recorded habitat composition and its robustness to contributor turnover. (**a**) Share of coded records in each habitat class by period, with the percentage-point change at right; denominators are the 158 and 103 records carrying a habitat description. (**b**) Test statistic for two single-degree-of-freedom period contrasts, unstratified and after Mantel–Haenszel stratification by province and by contributing institution; the dashed rule marks the critical value χ^2^_0.05_ = 3.84.

**Figure 4:**
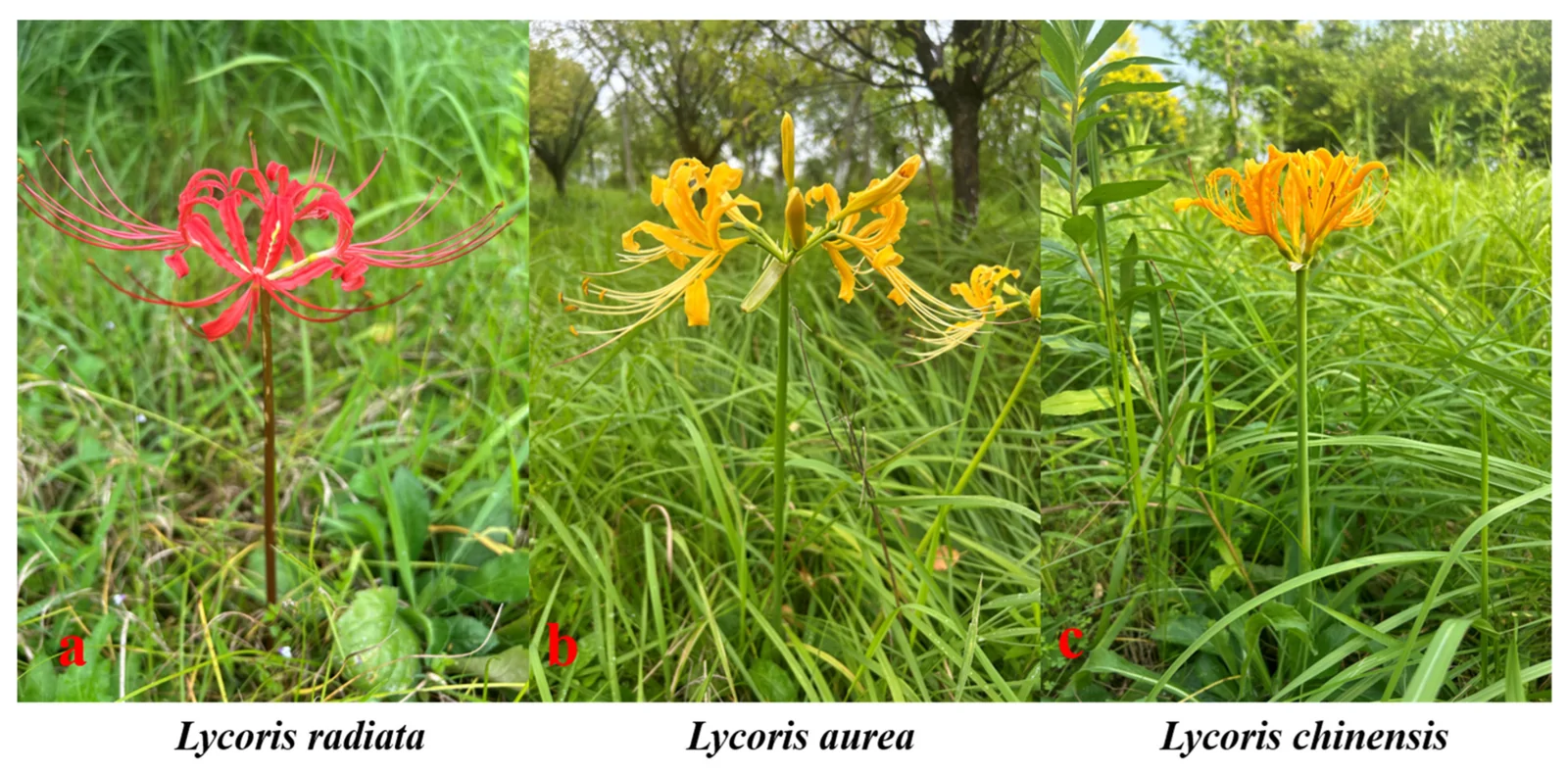
The three *Lycoris* species examined in this study, shown for morphological reference. (**a**) *L. radiata*, with deep-red, strongly recurved tepals and long-exserted stamens; (**b**) *L. aurea*, with a golden-yellow perianth; (**c**) *L. chinensis,* a flowering clump of *L. radiata*. All plants are cultivated accessions in the living collection of the Zhejiang Institute of Landscape Plants and Flowers, Zhejiang Academy of Agricultural Sciences, Xiaoshan District, Hangzhou 311251, Zhejiang, China. The photographs illustrate floral morphology only; they do not depict occurrence sites, and no habitat class was assigned or verified from them. Photographs by L.M.

**Table 1:** Voucher occurrence records by species and study period.

Species	2010–2015, *n*	2016–2020, *n*	Change, *n*	Change, %
*L. radiata*	113	58	−55	−48.7
*L. aurea*	63	46	−17	−27.0
*L. chinensis*	13	4	−9	−69.2
**Total**	**189**	**108**	**−81**	**−42.9**

*Note.* Records retrieved from the Chinese Virtual Herbarium and assigned to a period from the collection date on the specimen label. Counts represent available records, not abundance, occupancy, or population size. Period × species: χ^2^ = 3.258, df = 2, *p* = 0.196, Cramér’s V = 0.105, no expected count below five. After collapsing 56 duplicate gatherings the corresponding totals are 131 and 78 (χ^2^ = 3.046, df = 2, *p* = 0.218).

**Table 2:** Contributor turnover and stratified tests of the period × habitat association.

Comparison	χ^2^	df	*p*	Permutation *p*	Cramér’s V
Period × contributing institution (30 herbaria)	89.900	29	<0.001	<0.0001	0.550
Period × institution (5 largest vs. others)	25.734	5	<0.001	—	0.294
Period × province (17 units)	42.818	16	<0.001	0.0001	0.380
Institution × recorded habitat	349.318	138	<0.001	0.0001	0.472
Province × recorded habitat	341.224	90	<0.001	<0.0001	0.467
Period × Forest/Woodland, unstratified	13.828	1	<0.001	—	—
Period × Forest/Woodland, stratified by province	8.574	1	0.003	—	—
Period × Forest/Woodland, stratified by institution	6.250	1	0.012	—	—
Period × Grassland, unstratified	1.513	1	0.219	—	—
Period × Grassland, stratified by province	0.027	1	0.871	—	—
Period × Grassland, stratified by institution	0.683	1	0.409	—	—

*Note.* The first five rows are Pearson chi-square tests of independence; Cramér’s V is √(χ^2^/[*n*·min(r − 1, c − 1)]). The last six rows are single-degree-of-freedom tests of one habitat contrast against all other classes; the unstratified rows are Pearson tests, and the stratified rows are Mantel–Haenszel tests with continuity correction, stratified by province or by contributing institution. Strata falling entirely within one period or one habitat category carry zero variance and do not enter the statistic; 9 of 17 provinces and 13 of 30 institutions are informative for the Forest/Woodland contrast. Permutation *p* is a Monte Carlo permutation *p*-value from 20,000 replicates, reported for every table containing an expected count below five and shown as an em dash where none falls below five or the test is a Mantel–Haenszel contrast. The institution × habitat and province × habitat tables are sparse (155/168 and 96/112 expected counts below five) and their statistics indicate the magnitude of association rather than a precise inferential result. A contrast that remains significant under stratification cannot be attributed solely to turnover in the contributors.

**Table 3:** Recorded habitat composition of voucher records in the two study periods.

Recorded Habitat Class	2010–2015, *n* (%)	2016–2020, *n* (%)	Change, pp
Forest/Woodland	71 (44.9)	23 (22.3)	−22.6
Urban/Residential	14 (8.9)	21 (20.4)	+11.5
Other	32 (20.3)	22 (21.4)	+1.1
Grassland	23 (14.6)	21 (20.4)	+5.8
Shrubwood/Sparsewood	15 (9.5)	13 (12.6)	+3.1
Dry land	1 (0.6)	3 (2.9)	+2.3
Paddy land	2 (1.3)	0 (0.0)	−1.3
**Total**	**158 (100.0)**	**103 (100.0)**	**—**

*Note.* Classes were coded from collectors’ original free-text habitat descriptions; 36 of the 297 records carry no description and are excluded, so period denominators are 158 and 103. Percentages are within period; pp, percentage points; changes use unrounded proportions. Period × habitat, seven classes: χ^2^ = 20.308, df = 6, *p* = 0.002, Cramér’s V = 0.279, with 4 of 14 expected counts below five; permutation *p* = 0.002 from 20,000 replicates. Three-class grouping (Forest/Woodland, Grassland, Other): χ^2^ = 13.829, df = 2, *p* < 0.001, Cramér’s V = 0.230, no expected count below five. Habitat classes were assigned by a single coder against the rubric fixed in advance (Table 4 and Table S5); every original description and its assigned class are listed in Excel Table S1.

**Table 4:** Definitions of the seven recorded habitat classes.

Recorded Habitat Class	Definition Applied
Forest/Woodland	The plant’s immediate context is a stand of trees or its margin or understory: closed or open forest, plantation, bamboo stand, secondary woodland, forest edge, or forest understory.
Shrubwood/Sparsewood	The plant’s immediate context is a shrub layer or sparse woody scrub. Takes precedence over Forest/Woodland where both a shrub term and a tree term appear, because the shrub layer is the stratum the plant occupies.
Grassland	The plant’s immediate context is herbaceous vegetation with no woody stratum named: grassland, grassy slope, meadow, or a grassy valley or riverside surface.
Dry land	Cultivated non-irrigated farmland or its margin (dry field, upland crop field), distinguished from Grassland by an explicit cultivation term.
Paddy land	Irrigated rice field or its margin.
Urban/Residential	A built or actively managed human element is the plant’s immediate context: roadside or path verge, park, garden or managed planting, dam or embankment, village or settlement margin, or scenic-area planting. Takes precedence over every vegetation class.
Other	No vegetation type and no land-use element can be resolved from the description; the description records only landform, substrate, hydrology or ecological traits, or records nothing about the site.

Note: The seven classes are those of the national land-use classification of China and were adopted, rather than defined for this study, so that the results remain comparable with an earlier five-year analysis that used the same scheme [35]. Each of the 133 distinct free-text habitat descriptions was assigned to exactly one class. The six ordered rules of application, together with verbatim representative source phrases and their record counts, are given in Table S5; every original description and its assigned class are released in Excel Table S1. Following the convention stated in Section 4.5, habitat-class names are capitalized when they label the seven classes defined here, and lower case when the same words are used generically.

## Data Availability

The record-level data supporting every result in this article are provided as Table S1.

## Supplementary Materials

The following supporting information can be downloaded at: https://www.mdpi.com/article/10.3390/plants15192933/s1, Methods S1: Record retrieval, coordinate status, gathering identification, habitat coding, and statistical analysis; Table S1: Record-level table for the 297 voucher records, giving specimen identifiers, contributing herbarium, collection date and period, locality, coordinates and coordinate status, collector, the original habitat description, the assigned habitat class, and the gathering group; Table S2: Records by contributing herbarium and study period (2010–2015 vs. 2016–2020). Institution codes follow Index Herbariorum; the note reports the period × institution chi-square test; Table S3: Records by province and study period across 17 provincial-level units. The note reports the period × province chi-square test; Table S4: Coded habitat class by species and study period (n = 261 records with a habitat description). Species-level counts are provided for audit only; Table S5: Coding rubric used to assign the 133 distinct collector habitat descriptions to seven land-use classes, with the rules of application and representative verbatim descriptions; Table S6: Full summary of statistical tests: Pearson chi-square with Monte Carlo permutation *p*-values, Cramér’s V, stratified Mantel–Haenszel tests, and the duplicate-collapsed robustness tests; Table S7: Records by collection year and species. The decline after 2015 is consistent with digitization lag and does not indicate a population trend; Table S8: Coordinate status by species (valid decimal, converted from DMS, partial, missing, or 0,0 placeholder). Only the 214 records with valid or converted coordinates carry usable positions; Table S9: Data dictionary defining each field in Table S1.; and Excel Table S1 (Record-level table for the 297 voucher records (CSV file). Each row gives the specimen identifiers, contributing herbarium, collection date and period, locality, coordinates and coordinate status, collector, the collector’s original habitat description, the assigned habitat class, and the gathering group), a 297-row record-level table released as a separate CSV file, with institution code, catalog number, collection date, collector, locality, coordinates and explicit coordinate-status flags, original habitat text, coded habitat class, and gathering identifiers.
