# Supplementary material for: Contributor Turnover Confounds Temporal Comparison of Recorded Habitats: A Voucher-Based Case Study of Three Lycoris Species in China from 2010 to 2020
Source: Plants (Basel). 2026 Sep 25;15(19):2933. doi: 10.3390/plants15192933 (PMC13666690; doi:10.3390/plants15192933)
Supplement: Supplementary file 1 [file plants-15-02933-s001.zip › plants-4550684_supplementary_reordered.pdf]

Supplementary Material

Contributor Turnover Confounds Temporal Comparison of Recorded Habitats: A Voucher-Based Case Study of Three *Lycoris* Species in China from 2010 to 2020

Supplementary contents

Supplementary Methods S1. Record retrieval, coordinate status, gathering identification, habitat coding, and statistical analysis.

Table S1. Record-level table for the 297 voucher records (supplied as a separate CSV file).

Tables S2–S9. Contributing herbaria, provincial distribution, habitat by species, coding rubric, full test summary, records by collection year, coordinate status, and data dictionary.

Supplementary Methods S1

S1.1 Record retrieval

Records were retrieved from the Chinese Virtual Herbarium (https://www.cvh.ac.cn, accessed on 20 November 2025). The search was restricted to *Lycoris radiata*, *L. aurea*, and *L. chinensis*, to physical specimen records rather than image-only or literature entries, and to collection dates between 5 September 2010 and 20 September 2020. Records annotated as cultivated or as originating from botanical-garden or nursery material were excluded. No record was removed after retrieval. Species determinations were accepted as recorded by the contributing herbaria and were not independently revised.

S1.2 Coordinate status

Coordinates were retained only where the label values could be parsed to decimal degrees. Of the 297 records, 208 carry decimal-degree coordinates and 6 were converted from degrees–minutes–seconds (214 usable positions); 80 are missing, 2 partial, and 1 a 0,0 placeholder. All 214 positions fall inside the terrestrial bounding box of China and are consistent with the administrative unit named on the label. No record carries a coordinate-uncertainty radius or a statement of how the position was determined, so the coordinates were used only for display and for describing provincial distribution.

S1.3 Gatherings and duplicates

Records sharing collector, collection date, and locality were flagged as belonging to a single gathering; such records are predominantly herbarium duplicates of one collecting event distributed among institutions. In total 144 records (48.5%) fall into 56 gatherings. All primary analyses retain the flagged records, and every test was repeated on a collapsed dataset in which each gathering contributes one record ($n = 209$).

S1.4 Habitat coding

Collector habitat descriptions are free text and comprise 133 distinct strings. Each description was coded into one of seven classes by a single author, following the written rubric reproduced as Table S5, which was agreed before coding began. No independent second coding was performed, so no inter-coder agreement statistic is available; every original description and its assigned class are released in Table S1 so that the coding can be checked and repeated. The 36 records without a habitat description were excluded from habitat analyses, leaving 261 coded records with period denominators of 158 and 103.

S1.5 Statistical analysis

Pearson chi-square tests of independence were used for period × species, period × province, period × contributing institution, and period × coded habitat. Cramér's V was calculated as $V = \sqrt{\chi^2 / [n(k - 1)]}$, where χ^2 is the Pearson chi-square statistic for the table, n is the number of records contributing to that table, and k is the smaller of the number of rows and the number of columns. Because contributor composition changed between periods and both contributor variables are strongly

associated with recorded habitat, the period × habitat comparison was repeated under stratification using Mantel–Haenszel tests with continuity correction, for Forest/Woodland versus all other classes and for Grassland versus all other classes, stratifying in turn by province and by contributing institution. Every chi-square test computed on a record-level table containing an expected count below five was repeated as a Monte Carlo permutation test with 20,000 replicates, and the permutation p-value is reported alongside the asymptotic one in Table S6; the duplicate-collapsed tests are reported as a robustness check with asymptotic values only. Strata falling entirely within one period or one habitat category carry zero variance and do not enter a Mantel–Haenszel statistic; 9 of 17 provinces and 13 of 30 contributing institutions are informative for the Forest/Woodland contrast. All analyses and figures were produced in R.

Supplementary Tables

Table S2. Records by contributing herbarium and study period.

Institution code	2010–2015, n	2016–2020, n	Total
GZTM	30	25	55
IBK	10	20	30
GXMG	21	8	29
CSH	17	3	20
QFNU	16	1	17
CSFI	14	2	16
KUN	6	8	14
JIU	9	4	13
JJF	9	3	12
HIB	2	10	12
NAS	11	0	11
IMC	7	2	9
IBSC	2	6	8
GYBG	0	7	7
CDBI	4	2	6
PE	6	0	6
WUK	4	0	4
SYS	3	1	4
JXCM	4	0	4
HEAC	2	1	3
CZH	0	3	3
GXMI	3	0	3
JXU	2	1	3
IMDY	2	0	2
BJFC	1	0	1
GZAC	1	0	1
HENU	1	0	1
LBG	1	0	1
ZM	1	0	1
ZY	0	1	1
**Total**	**189**	**108**	**297**

Note. Institution codes follow Index Herbariorum. Thirty herbaria contributed records. Period × institution: $\chi^2 = 89.900$, df = 29, $p < 0.001$, Cramér's V = 0.550, with 40 of 60 expected counts below five; the same comparison with the five largest contributors against all others gives $\chi^2 = 25.734$, df = 5, $p < 0.001$ with no expected count below five.

Table S3. Records by province and study period.

Province	2010–2015, n	2016–2020, n	Total
Guizhou	33	33	66
Guangxi	36	28	64
Jiangxi	27	11	38
Hunan	24	6	30
Hubei	20	4	24
Henan	6	9	15
Sichuan	12	2	14
Anhui	6	6	12
Chongqing	7	2	9
Shanghai	6	0	6
Jiangsu	4	0	4
Fujian	1	3	4
Gansu	4	0	4
Zhejiang	0	3	3
Yunnan	2	0	2
Guangdong	0	1	1
Shaanxi	1	0	1
**Total**	**189**	**108**	**297**

Note. Seventeen provincial-level units are represented. Period × province: $\chi^2 = 42.818$, df = 16, $p < 0.001$, Cramér's V = 0.380, with 18 of 34 expected counts below five. Shanghai, Jiangsu, and Gansu contributed records only in the earlier period; Zhejiang and Guangdong only in the later one.

Table S4. Coded habitat class by species and study period.

Species	Period	Forest	Urban	Other	Grass	Shrub	Dry	Paddy	Total
L. radiata	2010–2015	39	13	18	16	8	1	2	97
L. radiata	2016–2020	12	11	8	16	5	3	0	55
L. aurea	2010–2015	23	1	11	7	7	0	0	49
L. aurea	2016–2020	10	10	11	5	8	0	0	44
L. chinensis	2010–2015	9	0	3	0	0	0	0	12
L. chinensis	2016–2020	1	0	3	0	0	0	0	4
**Total**	**—**	**94**	**35**	**54**	**44**	**28**	**4**	**2**	**261**

Note. Forest, Forest/Woodland; Urban, Urban/Residential; Grass, Grassland; Shrub, Shrubwood/Sparsewood; Dry, Dry land; Paddy, Paddy land. Records without a habitat description are excluded. Species-level tables are too sparse for separate inference and are provided for audit only.

Table S5. Coding rubric for collector habitat descriptions.

The seven classes are those of the national land-use classification of China, adopted so that results remain comparable with an earlier five-year analysis that used the same scheme. The rubric below was agreed before coding began and is reproduced in full so that every coding decision can be audited and repeated. Each of the 133 distinct habitat descriptions was assigned to exactly one class by applying the following rules in order.

Rules of application

1. Code the plant's immediate context as described by the collector, not the wider landscape setting. Terms of landform (hillside, valley, mountain area), substrate (calcareous soil, sandy soil), hydrology (streamside, beside a gully) and ecological trait (sun-loving, mesic, wild plant) are treated as modifiers and never determine the class on their own.

2. If a built or actively managed human element is named as the immediate context — roadside or path verge, park, garden, managed planting, dam, embankment, village or settlement margin — assign Urban/Residential, irrespective of any vegetation term also present.

3. Otherwise, if cultivated land is named, assign Paddy land for irrigated rice field and Dry land for other cultivated field.

4. Otherwise, assign the vegetation stratum the plant occupies. Where both a shrub term and a tree term appear, Shrubwood/Sparsewood takes precedence over Forest/Woodland; where both a herbaceous term and a woody term appear, the woody class takes precedence.

5. If no vegetation and no land-use element can be resolved under rules 2 to 4, assign Other.

6. A record carrying no habitat description at all is not coded and is counted among the 36 records without a description; a description that is present but records nothing identifiable about the site is assigned to Other.

Class definitions

Class	Definition applied	Representative source phrases (n records)
Forest/Woodland	The plant's immediate context is a stand of trees or its margin or understory: closed or open forest, plantation, bamboo stand, secondary woodland, forest edge, or forest understory. Applied when a tree-stratum term is the most specific vegetation term present and no rule 1–3 element applies.	forest edge (15); broadleaved forest (8); forest understory (8); hillside forest understory (4); valley forest edge (4); mixed conifer-broadleaved forest (3); in a Moso bamboo forest (2); mountain area; open woodland (2); subtropical evergreen broadleaved forest; forest edge (1)
Shrubwood/Sparsewood	The plant's immediate context is a shrub layer or sparse woody scrub. Takes precedence over Forest/Woodland when both a shrub term and a tree term appear, because the shrub layer is the stratum the plant occupies.	shrubland (7); shrubland; forest understory; calcareous soil (3); mountain area; shrubland (1); shrubland; forest edge; occasional wild (1); shrubland; sunny slope; yellow-brown soil (1); valley; loam (red soil); shrubland (1)
Grassland	The plant's immediate context is herbaceous vegetation with no woody stratum named: grassland,	grassland (7); grassland; mesic; sun-loving; moderate-temperature; acidic soil; wild plant (3); riverside at the foot of a hill grassland (2); in

Class	Definition applied	Representative source phrases (n records)
	grassy slope, meadow, or a grassy valley or riverside surface.	river-valley grassland (1); in valley grassland or cliff grassland (1); sunny site; grassy slope (1)
Dry land	Cultivated non-irrigated farmland or its margin (dry field, upland crop field). Distinguished from Grassland by an explicit cultivation term.	field margin (3); grassy field margin at the foot of a hill (1)
Paddy land	Irrigated rice field or its margin.	paddy-field margin (2)
Urban/Residential	A built or actively managed human element is the plant's immediate context: roadside or path verge, park, garden or managed planting, dam or embankment, village or settlement margin, or scenic-area planting. Takes precedence over every vegetation class (rule 2), because the defining feature of the site is human modification and maintenance.	roadside on a hillside (4); roadside (3); park (2); shaded site; roadside (2); grassland; roadside; yellow-brown soil; sparse wild (2); on a dam (5); open ground beside a road (3); on an earth bank (2); small scenic woodland near a village (1); shrubland; roadside; calcareous soil (1)
Other	No vegetation type and no land-use element can be resolved from the description. Includes descriptions that record only landform, substrate, hydrology or ecological traits, and descriptions that record nothing about the site.	hillside (10); streamside (7); waterside (4); xeric; sun-loving; moderate-temperature; wild plant (3); other (3); limestone substrate (1); rock crevice on a hillside (1); on rock; with water present (1); not recorded (1)

Note. Representative phrases are quoted verbatim from the collectors' descriptions as translated in Table S1; the number in parentheses is the number of records carrying that description. The coding was carried out by a single author against the rubric above; no independent second coding was performed, so no inter-coder agreement statistic is available (Section 4.5).

Table S6. Full summary of statistical tests.

Test	χ^2	df	p	Permutation p	Cramér's V	Expected < 5
Period × species	3.258	2	0.196	—	0.105	0/6
Period × species (gatherings collapsed, n = 209)	3.046	2	0.218	—	0.121	1/6
Period × province	42.818	16	< 0.001	0.0001	0.380	18/34
Period × institution (30 herbaria)	89.900	29	< 0.001	< 0.0001	0.550	40/60
Period × institution (5 largest vs. others)	25.734	5	< 0.001	—	0.294	0/12
Period × habitat, 7 classes	20.308	6	0.002	0.002	0.279	4/14
Period × habitat, 3 classes	13.829	2	< 0.001	—	0.230	0/6
Period × habitat, 7 classes (gatherings collapsed, n = 178)	9.371	6	0.154	—	0.229	4/14
Period × Forest/Woodland, unstratified (gatherings collapsed, n = 178)	2.173	1	0.140	—	—	0/4
Institution × habitat	349.318	138	< 0.001	0.0001	0.472	155/168
Province × habitat	341.224	90	< 0.001	< 0.0001	0.467	96/112
Period × Forest/Woodland, unstratified	13.828	1	< 0.001	—	—	0/4
Period × Forest/Woodland, MH by province	8.574	1	0.003	—	—	—
Period × Forest/Woodland, MH by institution	6.250	1	0.012	—	—	—
Period × Grassland, unstratified	1.513	1	0.219	—	—	0/4
Period × Grassland, MH by province	0.027	1	0.871	—	—	—
Period × Grassland, MH by institution	0.683	1	0.409	—	—	—

Note. MH, Mantel–Haenszel test with continuity correction. Permutation p is a Monte Carlo permutation p-value from 20,000 replicates, computed for every record-level table containing an expected count below five; an em dash marks tests for which no expected count falls below five, Mantel–Haenszel contrasts, and the duplicate-collapsed robustness test, which is reported with its asymptotic value only. Cramér's V is not reported for single-degree-of-freedom contrasts. Tables with a high proportion of small expected counts are marked accordingly and their statistics should be read as indicating the magnitude of association rather than as precise inferential results.

Table S7. Records by collection year and species.

Year	*L. radiata*	*L. aurea*	*L. chinensis*	Total
2010	3	2	0	5
2011	15	5	0	20
2012	18	13	1	32
2013	15	15	2	32
2014	29	13	10	52
2015	33	15	0	48
2016	24	10	0	34
2017	10	5	0	15
2018	10	12	0	22
2019	6	18	1	25
2020	8	1	3	12
**Total**	**171**	**109**	**17**	**297**

Note. Record counts peak in 2014–2015 and decline thereafter. Because recently collected specimens are least likely to have been accessioned, databased, and published to an aggregator at the time of retrieval, this profile is consistent with digitization lag and should not be read as a population trajectory.

Table S8. Coordinate status by species.

Species	Valid decimal	Converted from DMS	Partial	Missing	0,0 placeholder	Total
L. radiata	118	0	0	53	0	171
L. aurea	82	6	2	19	0	109
L. chinensis	8	0	0	8	1	17
**Total**	**208**	**6**	**2**	**80**	**1**	**297**

Note. Only valid_decimal and converted_dms records (n = 214) carry usable positions. All 214 fall inside the terrestrial bounding box of China and are consistent with the administrative unit named on the label.

Table S9. Data dictionary for Table S1.

Field	Definition
Record_ID	Stable identifier assigned in the cleaned table; species-specific prefix.
Original_occurrence_ID	Identifier from the source portal, retained for traceability.
Species	Scientific name as determined by the contributing herbarium.
Institution	Index Herbariorum code of the holding institution.
Catalog_number	Catalog or accession number of the specimen.
Period	Study period assigned from the collection date.
Collection_date	Collection date from the specimen label; ISO date where valid.
Province / County_or_district / Locality	Administrative unit and locality text, romanized in untoned Mandarin pinyin.
Longitude_dd / Latitude_dd	Decimal-degree coordinates; blank when missing, partial, invalid, or a 0,0 placeholder.
Coordinate_status	valid_decimal, converted_dms, partial, missing, or zero_placeholder.
Elevation_m	Elevation or elevation range in metres as recorded.
Collector(s)	Collector name or collecting team as recorded.
Habitat	Collector's original free-text habitat description.
Coded_habitat	Habitat class assigned under the rubric in Table S5; blank where no description exists.
Gathering_group	Identifier shared by records of one collecting event (herbarium duplicates).
Data_quality_note	Concise summary of the quality rules triggered by the record.

Note. No record was removed from Table S1. Specimen data remain subject to the terms of use of the contributing herbaria.
